# Theoretical musings

**DOI:** 10.7554/eLife.60703

**Published:** 2020-08-26

**Authors:** Eve Marder

**Affiliations:** Volen Center and Biology Department, Brandeis UniversityWalthamUnited States

**Keywords:** Living Science, theory, mathematical modeling, peer review

## Abstract

There is more to theory in biology than replicating the results of experiments – the best theory papers help experimentalists to identify which of their results might be general and to plan a path through the maze of all possible future experiments.

I have always thought of theory in biology as disciplined dreaming. The discipline comes from the challenge of creatively marrying the rules of mathematics and physics with what is known of fundamental biological principles. In biology, we face problems that seem inexplicable, or at least deeply puzzling. Theoretical studies can lead the way to explore potential explanations for these problems. As an experimentalist, I have been grateful that computational models can free us from the idiosyncrasies of the particular system we study. I learned this many years ago when I asked Tom Kepler, then a postdoc, to model the results of experiments on electrically coupled oscillators that Scott Hooper and I had published several years earlier: we had found that an isolated oscillatory neuron was faster when its electrically coupled neighbors were removed (Hooper and Marder, 1987). Tom’s first simulations showed the exact opposite and I told him he must have made a mistake! He was rightfully miffed, but returned shortly with simulations which showed that both results were possible, depending on the ratio of the inward and outward currents in the model (Kepler et al., 1990).

For me, the deep take-home message of this exercise was that I had no idea that there were two cases: that an oscillator, depending on the details of how it is built, could go either faster or slower when depolarized! Because of the specific properties of the neurons we had initially studied, we had seen only one of the two possible cases and mistakenly thought that result was general. Several years later, now knowing that both cases were possible, we recognized that a different cell type showed the second case (Weimann et al., 1993). Thus, for me as a biologist, theory is valuable because it enables us to ask which of our results are likely to be general and which depend on the particular characteristics of our experimental system.

We can also use theory to ask the following question: how might a biological system solve a particular problem? In the early 1990s many neuroscientists were struck by how difficult it was to 'tune' neuronal models – that is, to find parameters that would, at least loosely, replicate the biological systems they were meant to represent. On the other hand, biological neurons find good solutions, so clearly they were not randomly sampling parameter space, but had rules or mechanisms that allowed them to find such solutions. But how?

It was already clear that neurons could maintain stable properties for many years, despite the continuous turnover of membrane proteins. Our first models to tackle this problem of feedback regulation of intrinsic excitability, or homeostatic regulation of neuronal excitability, suggested that a simple negative feedback system with an activity sensor that controlled channel density, akin to other homeostatic processes that control temperature or blood pressure, might provide a plausible explanation (LeMasson et al., 1993). Almost thirty years later, there have been thousands of studies in an intellectual lineage that follows that initial framing of the question (Turrigiano, 2017). The initial model was useful because it drew attention to a biological conundrum in a concise manner, and because it triggered investigators to do experiments they might not have otherwise done, not because it was biologically correct in its details.

Many others have written cogently about the importance of theory in biology (see, for example, Goldstein, 2018). However, my reasons for writing this article are not just to extol the virutes of theory, but to explore the additional challenges that theory manuscripts and grant applications seem to experience during peer review. You might imagine that theorists would be free spirits, letting their imaginations roam free to conjure and appreciate interesting hypotheses that can drive new ways of thinking about difficult biological problems. Instead, I am struck that theorists (as well as experimentalists) – when acting as reviewers – seem to often ask for direct connection to known biological data, and are frequently critical of theory papers. Why might this occur?

**Figure 1:**
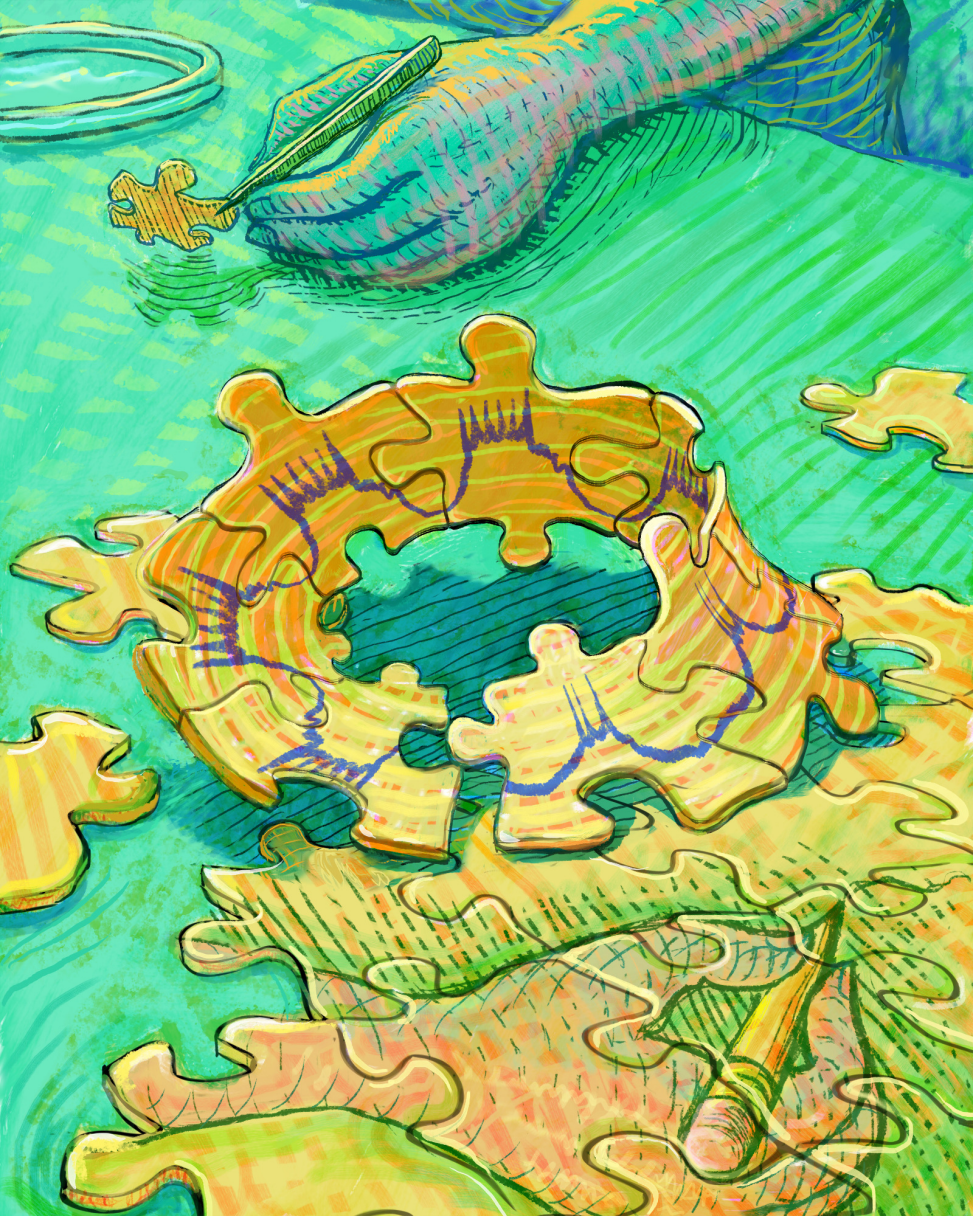
"A well-designed theoretical study can help an experimentalist to work out which features of any set of results likely approach a new general principle".

Illustration: Ben Marder.

Experimentalists know how difficult it can be to collect data if they don't have the necessary tools, or if the experiment would take years to perform, so it is helpful when there is a theoretical study that points to a path through the maze of all possible future experiments. A well-designed theoretical study can also help an experimentalist to work out which features of any set of results likely approach a new general principle: a theory that does no more than replicate an existing experimental data set is unlikely to help with this endeavor.

So why are so many smart reviewers constantly asking for data that corroborate the theory before the theory is even published?

So why are so many smart reviewers constantly asking for data that corroborate the theory before the theory is even published? I imagine that theorists are aware that one could construct and study innumerable problems that would provide little new insight. Thus, one can see this as a request for reassurance that the theoretical study at hand has the potential to illuminate a biological problem. I argue that the issue is not whether there are extant data, but whether the paper provides a novel concept, large or small, into a problem yet to be understood.

Ironically, a theory paper that 'solves' a difficult problem with a simple, transparent, and elegant solution can be easily devalued. It can take months or years of work to come to a result, which once understood, seems simple, and perhaps obvious. All of the underlying work and the multiple fruitless avenues of exploration may not show, once the answer is clear. And if the answer is a few simple differential equations or calculations, the reader might feel that they could have done the work in two hours, not remembering that until it had been done, no one knew the solution, or that it was an interesting problem.

Indeed, it can be easy to mistake the complexity of the mathematics in a theory paper for the importance of the new biological insight that ensues. Which isn’t to say that, for those capable of understanding the mathematics, that there isn’t deep value in work that requires innovative and sophisticated calculations. Likewise, a low-tech experiment might give more new insight than a more technologically demanding one. Again, it is important to not be fooled by complexity of method when evaluating the value of a scientific result.

It is rare for there to be a clear point at which any scientific problem is 'finished'. So the decision of what constitutes 'a paper' is always a judgment call. However, when reviewing manuscripts many of us are guilty of focusing on the paper we would have written rather than paying attention to the one written by the authors (Marder, 2020). This is especially true for theory papers that often seem to be evaluated in terms of what the reviewer might consider the most interesting path to take, rather than the path taken. Ironically, if the manuscript is successful, there should be endless new directions to pursue, some of which may look to be more interesting or prove to be more fruitful than those taken initially by the authors. But many of these paths will only appear to be interesting or potentially fruitful because of the insights provided by the authors in the submitted manuscript. We should be grateful for those theory papers that at once answer a question and pose new ones.

## Note

This essay is part of the Living Science collection.
